# Clinical characteristics of refractory mycoplasma pneumoniae pneumonia in children treated with glucocorticoid pulse therapy

**DOI:** 10.1186/s12879-021-05830-4

**Published:** 2021-01-28

**Authors:** Zhenli Zhu, Tongqiang Zhang, Wei Guo, Yaoyao Ling, Jiao Tian, Yongsheng Xu

**Affiliations:** 1 Tianjin Medical University, Tianjin Children’s Hospital (Children’s Hospital of Tianjin University), No.22, Qixiangtai Road, Heping District, Tianjin, 300070 China; 2 Department of Respiratory, Tianjin Children’s Hospital (Children’s Hospital of Tianjin University), Tianjin, People’s Republic of China; 3 Department of Pediatrics, Graduate School of Tianjin Medical University, Tianjin, 300074 People’s Republic of China; 4 Department of Respiratory, The Children’s Hospital of Tianjin (Children’s Hospital of Tianjin University), Tianjin, 300074 China

**Keywords:** Mycoplasma pneumoniae pneumonia, Children, Glucocorticoid

## Abstract

**Background:**

To observe the effect of corticosteroids in the treatment of children with refractory Mycoplasma pneumoniae pneumonia (RMPP) under different doses, to summarize the clinical features of children treated with glucocorticoid pulse therapy.

**Methods:**

The clinical data of 125 children with RMPP hospitalized in Tianjin Children’s Hospital from September 2018 to October 2019 were retrospectively analyzed. They were divided into two groups according to the dose of hormone. Compare the clinical features, laboratory findings, and imaging between the two groups, and use meaningful related indicators as ROC curves to find reference indicators for pulse therapy.

**Results:**

(1) The median age of the group II was older than that of the group I(*P* < 0.05). (2) We found more severe presentations, higher incidence of extra-pulmonary complications and more serious radiological findings in group II, which needed oxygen more often, higher the hormone, higher usage rate of gamma globulin, higher usage rate of bronchoscopy, and higher incidence of plastic bronchitis(*P* < 0.05). (3) WBC, CRP, LDH, FER, D-D dimer, APTT, TT, PCT, IL-6 and the percentage of neutrophils in peripheral blood in Group II were higher than those in Group I(*P* < 0.05). (4) In ROC curve analysis, CRP, LDH, FER, and neutrophils of leukocyte classification were independent related factors that could be used as valuable predictors of methylprednisolone pulse therapy for RMPP in children. The cut-off values were CRP44.45 mg/L, LDH590IU/L, FER411ng/L, and neutrophils in leukocyte classification were 73.75%, respectively.

**Conclusion:**

CRP ≥ 44.45 mg/L, LDH ≥ 590 IU/L, FER ≥ 411 ng/L, neutrophil≥73.75%, lung consolidation, and pleural effusion may be predictors that guide the treatment of RMPP with pulse dose of GC.

## Background

Mycoplasma pneumoniae (MP) is the main pathogens of community-acquired pneumonia (CAP) in children [1]. Mycoplasma pneumoniae pneumonia (MPP) is considered as a benign and self-limiting disease. However, it has been found that some children may progress to refractory Mycoplasma pneumoniae pneumonia (RMPP) after being treated with sufficient and long-term macrolide antibiotics in timely [2], which often leads to pulmonary necrosis and pleural effusion, which may not only be difficult to treat and costing, but also leave sequelae such as bronchiectasis, necrotizing pneumonia, bronchiolitis obliteransa and so on [3–7], thus affect the quality of life. Over-immune response of host plays an important role in the development of RMPP [8, 9]. Studies have confirmed the effectiveness of glucocorticoid (GC) in the treatment of RMPP [2, 10, 11]. GC are effective in the treatment of severe RMPP by down-regulating the cell-mediated immune response associated with lung injury during infection [12–15]. Therefore, on the basis of adequate anti-infective treatment, GC has attracted more and more attention [16, 17]. It is vital for clinicians to identify severe RMPP as early as possible and give pulse dose of hormone therapy. So, retrospective analysis was performed on 125 children with RMPP hospitalized in our hospital from September 2018 to October 2019. The purpose of this study was to compare the differences of clinical manifestations, laboratory data and imaging findings between two groups and to explore the predictive values of pulse therapy of RMPP.

## Methods

### Patients

This study selected 125 children with RMPP who were treated with different doses of GC at Tianjin Children’s Hospital from September 2018 to October 2019. All children meet the diagnostic criteria of MPP [18, 19]: (1) Symptoms and signs of pneumonia showed on admission, including fever, cough, abnormal lung auscultation and so on; (2) Chest imaging indicated pneumonia; (3) Positive results of serologic test. Included patients underwent anti-MP IgM titrations twice, both at the time of admission and upon discharge. Patients who showed either a seroconversion (negative to positive), or four-fold or greater increase in IgM titers and who had both symptoms with≥1:640 high titers [18]. RMPP was defined as a case with persistent fever, clinical and radiological deterioration after appropriate management with azithromycin for 7 days or more [2, 20]. Clinical and radiological deterioration were described as follows [10, 17]: aggravation of clinical signs was characterized by persistent fever, severe cough, dyspnea, etc. Radiological aggravation showed enlargement of pulmonary lesions, increased density, pleural effusion, and even necrotizing pneumonia and lung abscess.

The included had the following characteristics [19, 21]: (1) Meet the diagnostic criteria of MPP; (2) Meet the definition of RMPP; (3) Age ≤ 16 years old. The exclusion criteria included any of the following [19, 21]: (1) Patients who had a history of tuberculosis, bronchiectasis, or lung tumors; (2) Patients who had diseases such as severe malnutrition, unconsciousness, chronic cardiac and pulmonary disease, congenital disease or immunodeficiency; (3) Patients who received GC before admission; (4) Patients who were discharged within 8 h after admission.

### Study design

The 125 children were divided into two groups. Group I was given conventional dose methylprednisolone 2 mg/kg/day(< 200 mg/day) (*n* = 81), and group II was treated with methylprednisolone pulse therapy ≥200 mg/day(*n* = 44).

This study was approved by the ethics committee of the Tianjin Children’s Hospital (Approved No. of ethic committee: L2021–01). The ethics committee waived the need for written informed consent provided by participants due to the retrospective nature of the study, because all patient data were analyzed anonymously, and no additional informed consent was required.

### Hormone grouping [22–24]

Conventional dose was defined as intravenous infusion of methylprednisolone 2 mg/kg/day(< 200 mg/day) (or an equivalent dose of dexamethasone, hydrocortisone, prednisolone or betamethasone), and pulse therapy ≥200 mg/day methylprednisolone (or an equivalent dose of dexamethasone, hydrocortisone, prednisolone or betamethasone).

### Grouping [25]

All selected children were treated with routine dose of methylprednisolone intravenously within 48–72 h after admission. According to changes in body temperature, children were divided into conventional dose group and pulse dose group:1) Conventional dose group: after given the initial of methylprednisolone 2 mg/kg/day(< 200 mg/day), their body temperature returned to normal within 48 h, their imaging abnormality gradually improved, CRP returned to normal, and there was no recurrence in the process of hormone withdrawal. 2) Pulse therapy: initially given methylprednisolone 2 mg/kg/day, there was no significant decrease in heat peak within 48 h. Therefore, gradually increased the dose of methylprednisolone. When the dose of methylprednisolone was increased to 200 mg/day or more, their body temperature returned to normal within 48 h.

### Collection of clinical data

Collect data for each patient, including demographic characteristics, medical history, physical examination results, laboratory data, radiology results, hospital stay, fever time, etc. Record the laboratory data which were detected during hospitalization, including white blood cell (WBC) counts, neutrophil counts, and levels of C-reactive protein (CRP), lactate dehydrogenase (LDH), procalcitonin (PCT), alanine aminotransferase (ALT), aspartate aminotransferase (AST), etc [26]. Hypoxia was defined as any oxygen saturation measured by pulse oximetry in indoor air < 92% [27, 28].

### Statistical analysis

Statistical analyses were performed using SPSS software (version 22.0). Normal distribution data were expressed as mean ± SD (x ± s). Independent-Samples T-test or One-way ANOVA was used to process these data. The skewed distribution data were presented as the median values (P25, P75), and comparisons were made by the Mann-Whitney U-test. Chi-squared tests were used to compare numerical data, which was presented as rate or constituent ratio. Meanwhile, we use the laboratory indicators with significant differences as independent related risk factors to make the ROC curve, and use the area under the ROC curve (AUC) to reflect the accuracy of the diagnostic test. Take the point closest to the upper left corner of the ROC curve, which has the largest sum of sensitivity and specificity, as the optimal value of prediction. The difference was considered statistically significant at *P* < 0.05.

## Results

### Clinical characteristics

**Fig. 1:**
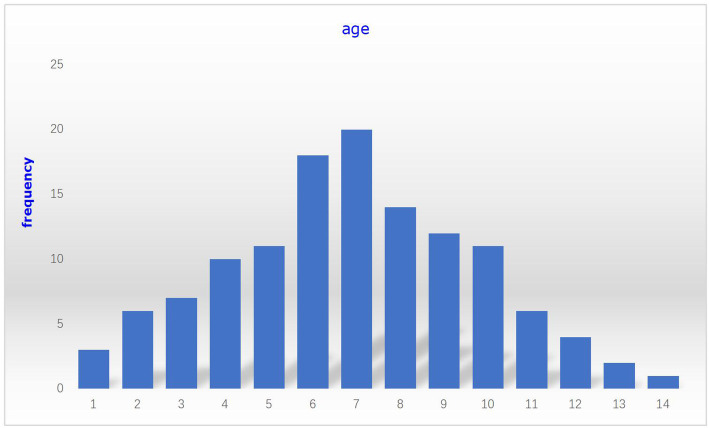
Age distribution of refractory Mycoplasma pneumoniae pneumonia patients

This study included 125 children. Children were divided into two groups according to GC dose. The age distribution of the subjects was shown in Fig. 1. The 81 patients in group I (36 females, 45 males) had the median age of 6.54 ± 3.03 years, and the median weight of 20.4(16.4, 28.9) kg; The 44 patients in group II (22 females, 22 males) had the median age of 7.61 ± 2.49 years, and the median weight of 27.2(20.0, 33.5) kg, which was shown in Table 1. Gender distribution was not differ between the two groups (*P* > 0.05). There were significant differences between the two groups in the age and weight (*P* < 0.05, *P* < 0.01).

**Table 1:** Clinical characteristic

Clinical information	Group I(81)	Group II (44)	*P*-value
General information			
Sex (female/male)	36/45	22/22	0.552
Age, years	6.54 ± 3.03	7.61 ± 2.49	0.031
Weight, kg	20.4 (16.4, 28.9)	27.2 (20.0, 33.5)	0.002
Clinical presentation n (%)			
Fever	81 (100%)	44 (100%)	1.00
Cough	75 (92.6%)	43 (97.7%)	0.891
Hypoxemia	6 (7.41%)	28 (63.6%)	0.000
respiratory failure	0	2 (4.55%)	0.041
Extra-pulmonary complications	18 (22.2%)	33 (75.0%)	0.007
Thromboembolism	2 (2.47%)	2 (4.55%)	0.613
total fever duration, days	11 (8, 13)	13 (11, 15)	0.000
preadmission fever duration, days	7 (5, 8)	6 (4, 7)	0.329
fever duration at the time of CS treatment^a^, days	1.95 ± 1.91	5.45 ± 2.76	0.000
Length of stay, days	8 (7, 10)	13.5 (11.3, 16)	0.000
Management			
Hormone dose, mg/kg/day	2 (1.5, 2)	10 (8.5, 10)	0.000
Gamma globulin, n (%)	9 (11.1%)	17 (38.6%)	0.000
Bronchoscopy, n (%)	57 (70.4%)	41 (93.2%)	0.000
Plastic bronchitis, n (%)	14 (17.3%)	29 (65.9%)	0.000

Data are presented as number (percentage), median (25th–75th percentile). Data are presented as mean ± SD (x ± s)

^a^The meaning of fever duration at the time of corticosteroids treatment is that fever days after starting corticosteroids therapy

### Laboratory findings

The laboratory values were shown in Table 2. The median values of Fg, PT, PLT, La, ALT and AST between the two groups were not statistically different (*P* > 0.05). WBC, CRP, LDH, FER, D-dimer, APTT, TT, PCT, IL-6 and the percentage of peripheral neutrophils in group II were higher, with a significant difference(*P* < 0.05).

**Table 2:** Laboratory characteristic

Laboratory information	Group I(81)	Group II (44)	*P*-value
White blood cell (× 10^9^/L)	9.00 (7.50, 11.2)	11.3 (9.20, 14.8)	0.001
Neutrophil,%	66.0 (48.0, 73.75)	81.6 (76.3, 87.0)	0.000
Lymphocytes,%	25.5 (16.85, 40.0)	12.1 (8.25, 15.0)	0.000
CRP, mg/L	36.9 (12.5, 67.2)	69.0 (54.0, 108)	0.000
LDH,IU/L	456 (345, 586)	594 (397, 776)	0.007
Fer, ng/L	255 (126, 345)	543 (321, 828)	0.000
D-D, mg/L	0.4 (0.2, 1.0)	1.15 (0.30, 3.28)	0.011
Fg, g/l	3.70 (3.31, 4.37)	3.84 (3.30, 4.72)	0.307
PT	11.9 (11.4, 12.4)	11.7 (11.0, 12.3)	0.147
APTT	30.8 (27.8, 34.3)	26.4 (24.0, 30.4)	0.000
TT	16.3 (15.5, 17.2)	17.1 (16.2, 17.8)	0.002
PLT	338 (269, 448)	294 (225, 408)	0.054
PCT, ng/ml	0.18 (0.10, 0.37)	0.33 (0.17, 0.55)	0.007
IL-6,pg/ml	26.0 (14.8, 45.9)	46.7 (25.1, 100.9)	0.001
La, mmol/L	2.81 (2.36, 3.36)	2.86 (2.35, 3.50)	0.781
AST,U/L	35 (27, 53)	39 (29, 53)	0.460
ALT,U/L	17 (12, 23)	21 (12.3, 51)	0.094

Data are presented as median (25th–75th percentile)

*WBC* White blood cell, Neutrophil Peripheral neutrophils, Lymphocytes Peripheral Lymphocytes, *CRP* C-reactive protein, *LDH* Lactic dehydrogenase, *Fer Ferritin*, *D-D D*-dimer, *Fg* Fibrinogen, *PCT* Procalcitonin, IL-6 Interleukin (IL)-6, *La* Lactic acid, *AST* Aspartate aminotransferase, *ALT* Alanine aminotransferase

### Imaging findings

Table 3 summarized the radiological findings in two groups. There were no differences in the incidence of atelectasis and pleural thickening between the two groups (25.0% versus 21.0, 68.2% versus 64.2%, *P* > 0.05). The incidence of pulmonary consolidation (93.2% versus 51.9%) and pleural effusion (45.5% versus 27.2%) in children with group II were higher than those in group I (*P* < 0.01, *P* < 0.05). Radiological findings were more severe in group II.

**Table 3:** Radiological features

Radiological features	Group I(81)	Group II (44)	*P*-value
Pulmonary consolidation, n (%)	42 (51.9%)	41 (93.2%)	0.000
Pleural effusion, n (%)	22 (27.2%)	20 (45.5%)	0.039
Lobar atelectasis, n (%)	17 (21.0%)	11 (25.0%)	0.607
Pleural thickening, n (%)	52 (64.2%)	30 (68.2%)	0.654

Data are presented as number (percentage)

### Treatment

All patients received macrolide therapy. Because the group II had more serious manifestations, higher incidence of extrapulmonary complications and more serious imaging findings, the group II’s the usage rate of gamma globulin, the usage rate of bronchoscopy, the incidence of plastic bronchitis were higher than the group I, with a significant difference (*P* < 0.05, *P* < 0.01, *P* < 0.01).

### Risk factors for RMPP of pulse dose GC

The ROC curves shows that CRP, LDH, FER, neutrophil percentage as independent risk factors for children with RMPP treated with pulse dose (Table 4). The cut-off values for CRP, LDH, FER, neutrophil percentage were set at 44.45 mg/L, 590 IU/L, 411 ng/L, 73.75 respectively. The sensitivity and specificity were respectively 55.0% & 85.0, 76.3% & 47.5, 86.4% & 68.2, and 75.0% & 90.0%.

**Table 4:** Predictive values of the independent correlation factors

Independent factors	Cutoff value	Sensitivity	Specificity	AUC	*P*-value	95%CI
Neutrophil, %	73.75	0.750	0.900	0.888	0.000	0.831–0.945
CRP, mg/L	44.45	0.550	0.850	0.736	0.000	0.648–0.825
LDH, IU/L	590.0	0.763	0.475	0.611	0.048	0.503–0.719
Fer, ng/L	411.0	0.864	0.682	0.814	0.000	0.736–0.892

*P* value: the AUC value of the independent factors compared to ROC curve reference value 0.5

*AUC* Area under the ROC curve, *Cut-off value* The value on the ROC curve is closest to the upper right to take maximum sensitivity and specificity

## Discussion

One of the main pathogens of CAP in children is Mycoplasma pneumoniae. More and more RMPP have been reported [10, 29–33]recently. Studies [34, 35] have shown that more than 90% of Mycoplasma pneumoniae infections in China are caused by drug-resistant strains. However, the latest research results of Sun et al. [36] have shown that the important cause of MP resistance to macrolide antibiotics was not only related to the irregular use of antibiotics, but also related to the epidemic genotype M4–5–7-2 of Mycoplasma pneumoniae. Through the comparison of genotypes and drug resistance between Chinese, American and Australian strains, it was reasonably explained from a new perspective that the high drug resistance rate in China and even in Asia is not all caused by the abuse of antibiotics, which is closely related to the regional differences in the epidemic genotypes of Mycoplasma pneumoniae [36]. Therefore, macrolides are still used in patients with MP in China. Only when macrolides are ineffective, antibiotics such as tetracyclines or fluoroquinolones can be used according to the condition [37–41]. Due to the influence of the pathogenesis, most of the RMPP will produce complications. The host’s excessive immune response plays a key role in the development of RMPP disease [8, 9], such as cytokines (including interleukin-2, interleukin-6 and interleukin-8). Over-expression and highly activated cells (including antigen presenting cells and T cells) mediated immune response etc [42]. GC can be used to down-regulate the related cell-mediated immune response and play an effective role in severe cases of MP infection [12–14]. Early control of lung injury caused by overactive immune response by non-specific adaptive immune cells is essential for reducing the incidence of severe MPP and preventing disease progression. Because the severity of RMPP is related to the immune response, and the effect of GC is dose-dependent, higher doses may be needed in patients with severe MPP [4, 43, 44].

A number of studies have revealed that humoral and cellular immune responses [45, 46]contribute to the pathogenesis of MP infection, providing a theoretical basis for the application of GC in RMPP. Studies have shown that the addition of GC on the basis of the conventional treatment has a definite effect on RMPP, which contributes to the control of the disease progression, the improvement of the condition and the reduction of sequelae [10, 16]. So, it is very important to study the application of GC in the treatment of RMPP [2, 16, 47].

So, in this retrospective research, 125 patients of RMPP were enrolled. Among them, there were 81 cases in group I, and 44 cases in group II. First of all, this study found that there was a statistical difference in age between group I and group II (6.54 ± 3.03, 7.61 ± 2.49, *P* < 0.05), which was similar to the previous studies [11, 17]. Children’s immune systems gradually mature with age. The more mature of immune system, it is more likely to have a strong inflammatory response to MP and produce too many inflammatory factors,which may lead to the deterioration of RMPP [17].

Secondly, the incidence of hypoxemia, extra-pulmonary complications and plastic bronchitis were higher in the group II than those in the group I (*P* < 0.05). Besides, the use rate of oxygen therapy, gamma globulin and bronchoscopy in the group II were higher(*P* < 0.05). The total fever days, hospital stay, fever days after hormone therapy in group II were significantly higher than in group I (*P* < 0.05). In addition, the study also found that WBC, CRP, LDH, FER, D-D dimer, APTT, TT, PCT, IL-6, ALT and the percentage of neutrophils in peripheral blood in group II were higher than in group I (*P* < 0.05). Finally, the incidence of pulmonary consolidation and pleural effusion were higher in group II(*P* < 0.05). The imaging findings may be related to the severity of the disease. The difference may is correlation to direct damage and immune inflammatory response [19]. So, if RMPP was not treated effectively, the disease maybe aggravated and the clinical process of disease maybe prolonged.

In order to study the clinical characteristics that can predict the severity of RMPP disease and guide the GC pulsed dose treatment, analyze statistically significant indicators by ROC curve. In ROC curve analysis, CRP, LDH, FER and white blood cell classification of neutrophils were helpful for identifying more severe RMPP patients. The optimal cutoff value were 44.45 mg/L,590 IU/L,411 ng/L and 73.75%, respectively. This study found that CRP 44.45 mg/L, LDH 590 IU/L, FER 411 ng/L, leukocyte classification neutrophil 73.75%, lung consolidation and pleural effusion may be important clinical features of use pulsed dose hormones to treat RMPP.

CRP is the most widely used acute phase inflammatory protein. CRP rises rapidly after inflammation stimulation, which value can reflect the development of the immune system. CRP levels in patients with acute infection, inflammation or trauma may increase in a short time. Chen et al. [19, 21]. showed that when CRP was 16.5 mg/L or higher, the sensitivity and specificity for diagnosing MPP with hypoxia were 74.7 and 77.2%, respectively. The cutoffs were less than that in our study. In our study, the optimal cutoff point for CRP was 44.45 mg/L, with a sensitivity of 55% and specificity of 85%.

LDH is an inflammatory marker. After cell damage, LDH is released into the serum and can be used to monitor tissue damage in many inflammatory processes. Studies have shown that LDH was related to many lung diseases [48, 49]. Serum LDH was a biomarker of RMPP severity [1, 11, 13, 19]. Lu et al. [50].reported that serum LDH can be used as a biomarker for predicting RMPP and evaluating whether to initiate corticosteroid therapy during the initial hospitalization of patients. Chen et al. [19, 21]. showed that when LDH was 417 IU/L or higher, the sensitivity and specificity for diagnosing MPP with hypoxia were 79.7 and 65.0%, respectively. In this study, the optimal cutoff for LDH was 590 IU/L, with a sensitivity of 76.3% and specificity of 47.5%, which was higher than that of previous studies [13, 19].

Elevated levels of ferritin may be positively correlated with the severity of inflammation, infection, renal failure and metabolic syndrome. In lung diseases, lung inflammation and tissue damage can lead to increased ferritin levels [51]. Kawamata et al. [12] showed that serum ferritin levels were positively correlated with the severity of children’s MPP, and ferritin may be a useful indicator for the initiation of glucocorticoid therapy for MPP. However, there is still no report about the correlation of ferritin in treatment of RMPP with pulse dose of GC. Choi et al. [52] reported that when ferritin was greater than or equal to 230 ng/mL, the sensitivity and specificity for diagnosing RMPP were 67 and 67%. In this study, the area under the curve for ferritin was 0.814 in the ROC curve analysis, which indicates that ferritin has a fair discriminative power in predicting the use of pulse dose to treat RMPP. The optimal cutoff for ferritin was 411 ng/mL, the sensitivity and specificity were 86.4 and 68.2%. The reasons for the difference in the studies are as follows: Firstly, it may be that the research object is RMPP, and the clinical manifestations are more serious; secondly, it may be that the research object contains unrecognized mixed infections.

Systemic GC can be considered for severe MPP with acute onset, rapid progression, especially for RMPP. However, there is no corresponding indicator for the use and timing of hormone dose. There are different opinions on the dosage of hormones in the existing article [11, 16]: You and Lee et al. [42]used intravenous infusion of methylprednisolone 10 mg/kg/day× 3 days for some patients who had failed oral treatment (dose reduction within 1 week). The clinical manifestations of all patients were significantly improved, and there were no related side effects. Lee [11] et al.treated 15 children with RMPP orally with prednisolone 1 mg/kg/day, and the dose was reduced after continuous use for 3–7 days, which has a significant therapeutic effect on children with RMPP. Luo et al. proved that oral prednisone (2 mg/kg/day) was more effective than azithromycin alone in children with RMPP. And Tamura [2] gave 6 patients with RMPP an intravenous drip of 30 mg/kg/day× 3 days methylprednisolone. The body temperature of all patients returned to normal within 14 h, and the clinical symptoms were significantly improved. They think that the combined use of hormone therapy can reduce the length of hospital stay and the occurrence of RMPP, and there is no adverse hormone response. The study implied that elder children are prone to more severe presentations, higher incidence of extra-pulmonary complications and more serious imaging. The study suggested that the severity of RMPP was related to host immune response, and the optimal values of CRP, LDH, FER and leukocyte classification neutrophils (CRP44.45 mg/L, LDH590IU/L, FER411ng/L, leukocyte classification neutrophils 73.75%), lung consolidation, and pleural effusion may be the valuable predictors of using methylprednisolone pulse therapy to treat RMPP.

This study indicated that in the treatment of RMPP, timely use of appropriate doses of GC can reduce the intensity of local inflammation, alleviates the immune reaction, and promote disease recovery. During the treatment of RMPP with GC, blood pressure, blood glucose, blood potassium and liver function should be monitored, notice the adverse reactions such as circulatory system and gastrointestinal bleeding, and pay attention to ECG monitoring during pulse dose treatment. And be sure to: ①the suitable time for treatment; ②exclude whether there are other infections or lesions; ③prevent the occurrence of double infection.

The study has some limitations. Firstly, retrospective research may have selection bias,which may need large sample, further prospective studies. Second, our hospital is a tertiary hospital with many severely ill patients. The uneven distribution of critically ill patients in this study has a certain impact on the experimental results. Thirdly, the source of patients is relatively single, and the research results may not be ideally suited for patients from other sources. To solve this problem, multi-regional research is required in the future. Fourth, some patients may be infected with other pathogens, but this pathogen has not been detected, and it may cause the deterioration of RMPP. Finally, the optimal value of risk factor obtained by ROC curve may have some limitations and only guide judgment to a certain extent. More clinical data should be accumulated and further verified in clinical work to obtain more accurate reference standards.

## Conclusion

This research shows that immune inflammatory response may play a vital role in the progression of RMPP. CRP ≥ 44.45 mg/L, LDH ≥ 590 IU/L, FER ≥ 411 ng/L, neutrophil≥73.75%, lung consolidation, and pleural effusion may be meaningful predictors that guide the treatment of RMPP with pulse dose of GC, which can reduce the incidence of severe RMPP and the occurrence of severe sequelae.

## Data Availability

The datasets used and/or analyzed during the current study are available from the corresponding author on reasonable request.

## Abbreviations

MP: Mycoplasma Pneumoniae
CAP: Community Acquired Pneumonia
RMPP: Refractory Mycoplasma pneumoniae pneumonia
PCR: Polymerase chain reaction
CRP: C-reactive protein
Fer: Ferritin
D-D: D-dimer
Fg: Fibrinogen
LDH: Lactate dehydrogenase
PCT: Procalcitonin
IL: Interleukin
La: Lactic acid
AST: Aspartate aminotransferase
ALT: Alanine aminotransferase
ROC: Receiver operating characteristic
